# Personal

**Published:** 1894-02

**Authors:** 


### Personal.

Dr. Joseph Price, of Philadelphia, has resigned as physician-in-charge of the Preston Retreat, and Dr. Richard C. Norris, associate editor of the *Annals of Gynecology and Pediatry*, has been elected his successor. It has been apparent to the friends of Dr. Price for some time that his resignation would soon become necessary, on account of the enormous demands upon his time made by his work in abdominal surgery. If he has no rival in this country in the latter field, it is also to his everlasting credit that he has made the Preston Retreat, during his six years of service, the most famous maternity in the world.

---

Dr. W. H. Myers and Dr. Miles F. Porter, of Fort Wayne, Ind., having been recommended for Pension Examiners by Hon. W. F.

McNagny, have been duly constituted a Pension Board. The Fort Wayne *Medical Magazine* comments approvingly on Mr. McNagny's judgment, as follows: "He has not only risen above mere party dictation, but has recognized honorable and progressive men in an honorable profession. Physicians everywhere should take this as an indication that they may exercise the rights of citizenship in a manner becoming the profession, and yet be selected for their worth rather than for their lack of it."

---

DR. EUGENE SMITH, of Detroit, according to rumor, has disposed of his Athol Springs hotel and the Spring House, located near Buffalo, to the Fresh Air Mission, of this city. It is anticipated that the Mission will convert the hotel into an Infants' Hospital for Summer use.

---

DR. LUCIEN HOWE, of Buffalo, was elected a member of the Ophthalmological Society of the United Kingdom (Great Britain and Ireland), on December 14, 1893. This exceptional honor, we believe, has been conferred in but one other instance on a resident of the United States.

